# Effects of AST-120 on mortality in patients with chronic kidney disease modeled by artificial intelligence or traditional statistical analysis

**DOI:** 10.1038/s41598-024-51498-6

**Published:** 2024-01-06

**Authors:** Chia-Lin Lee, Wei‑Ju Liu, Shang-Feng Tsai

**Affiliations:** 1 Division of Endocrinology and Metabolism, Department of Internal Medicine, Taichung Veterans General Hospital, Taichung, Taiwan; 2 Intelligent Data Mining Laboratory, Department of Medical Research, Taichung Veterans General Hospital, Taichung, Taiwan; 3 Department of Public Health, College of Public Health, China Medical University, Taichung, Taiwan; 4 School of Medicine, National Yang-Ming University, Taipei, Taiwan; 5 Department of Post-Baccalaureate Medicine, College of Medicine, National Chung Hsing University, Taichung, Taiwan; 6 Division of Nephrology, Taichung Veterans General Hospital, Taiwan, 160, Sec. 3, Taiwan Boulevard, Taichung, 407 Taiwan; 7 Department of Life Science, Tunghai University, Taichung, Taiwan

**Keywords:** Diseases, Medical research, Nephrology

## Abstract

Chronic kidney disease (CKD) imposes a substantial burden, and patient prognosis remains grim. The impact of AST-120 (AST-120) on the survival of CKD patients lacks a consensus. This study aims to investigate the effects of AST-120 usage on the survival of CKD patients and explore the utility of artificial intelligence models for decision-making. We conducted a retrospective analysis of CKD patients receiving care in the pre-end-stage renal disease (ESRD) program at Taichung Veterans General Hospital from 2000 to 2019. We employed Cox regression models to evaluate the relationship between AST-120 use and patient survival, both before and after propensity score matching. Subsequently, we employed Deep Neural Network (DNN) and Extreme Gradient Boosting (XGBoost) models to assess their performance in predicting AST-120's impact on patient survival. Among the 2584 patients in our cohort, 2199 did not use AST-120, while 385 patients received AST-120. AST-120 users exhibited significantly lower mortality rates compared to non-AST-120 users (13.51% vs. 37.88%, *p* < 0.0001) and a reduced prevalence of ESRD (44.16% vs. 53.17%, *p* = 0.0005). Propensity score matching at 1:1 and 1:2 revealed no significant differences, except for dialysis and all-cause mortality, where AST-120 users exhibited significantly lower all-cause mortality (*p* < 0.0001), with a hazard ratio (HR) of 0.395 (95% CI = 0.295–0.522). This difference remained statistically significant even after propensity matching. In terms of model performance, the XGBoost model demonstrated the highest accuracy (0.72), specificity (0.90), and positive predictive value (0.48), while the logistic regression model showed the highest sensitivity (0.63) and negative predictive value (0.84). The area under the curve (AUC) values for logistic regression, DNN, and XGBoost were 0.73, 0.73, and 0.69, respectively, indicating similar predictive capabilities for mortality. In this cohort of CKD patients, the use of AST-120 is significantly associated with reduced mortality. However, the performance of artificial intelligence models in predicting the impact of AST-120 is not superior to statistical analysis using the current architecture and algorithm.

## Introduction

The progression from any renal disease unresolved within 3 months ultimately leads to chronic kidney disease (CKD). CKD encompasses a diverse array of disorders characterized by both structural and functional impairment of the kidneys. These manifestations vary widely, with outcomes influenced by underlying causes and disease severity^1,2^. Recent years have seen numerous mechanisms proposed to elucidate the progression of CKD, and the global burden of disease report in 2017 revealed CKD as a significant contributor to mortality, causing 1.2 million deaths and ranking as the 12th leading cause of death worldwide^3^. All-age CKD mortality increased by 41.5% from 1990 to 2017^3^. A recent analysis estimated the global prevalence of CKD at 9.1% (697.5 million cases) in 2017^4^ , with Taiwan reporting a notably high national prevalence of 11.93%^5^. The progression of CKD carries severe consequences, including a heightened risk of mortality, end-stagerenal disease (ESRD), mineral bone disease, accelerated cardiovascular disease (CVD), and infections^6^. A cohort study involving 462,293 individuals^6^ revealed that patients with CKD had an 83% higher all-cause mortality (hazard ratio (HR) 1.83, 95% CI 1.73–1.93) and a 100% higher risk of CVD (HR 2.00, 95% CI 1.78–2.25). Unfortunately, there is a scarcity of medications available for halting CKD progression and reducing all-cause mortality.

Recent studies have reported renal benefits when applying Keto-analogue (Ketosteril) alongside a low or very low protein diet (LPD/VLPD)^7–9^*.* However, these studies did not find evidence of Ketosteril reducing all-cause mortality. In CKD, two major uremic toxins are Indoxyl Sulfate (IS) and p-Cresol (PC)^10^. IS originates from the metabolism of tryptophan, initially digested by intestinal bacteria into indole. This indole is subsequently metabolized into IS in the liver^11^ eventually excreted in the urine. On the other hand, PC is produced through the bacterial fermentation of tyrosine and phenylalanine^12^. Both IS and PC levels rise with declining renal function, further impairing kidney function1^13^. There is a strong correlation between estimated glomerular filtration rate (eGFR) and serum IS levels. IS is known to cause interstitial fibrosis and glomerulosclerosis^14^, contributing to CKD progression. Moreover, for CKD patients, there is a robust association between their plasma IS levels and the risk of vascular mortality^13^. PC also accumulates as renal function declines, and it can be used to predict mortality in patients undergoing hemodialysis^15^. A U.S. national prospective cohort^16^ study found that a higher PC concentration was associated with a higher risk of cardiovascular mortality (HR 1.62, 95% CI 1.17–2.25, *p* = 0.004). Therefore, the standard approach in CKD care involves the removal of IS and PC to mitigate CKD progression.

AST-120 (AST-120) is a spherical activated carbon with a diameter ranging from 0.2 to 0.4 mm. Its spherical shape enhances fluidity, potentially facilitating smoother passage through the gastrointestinal tract and better adsorption of IS and PC. AST-120 has received approval for halting CKD progression in several countries, including Japan (since 1991), Korea (since 2004), Taiwan (since 2007), and the Philippines (since 2010). A retrospective pair-matched study involving 560 patients^17^ found that AST-120 delayed the initiation of dialysis in CKD patients but had no effect on overall survival. In a randomized control trial^18^ the measured GFR dropped more in the control group than in the AST-120 group (-15% per year vs. -12% per year relative to the baseline value). Another long-term follow-up study^19^ reported a statistically significant improvement in the mean 1/serum creatinine in the AST-120 treatment group, along with an estimated delay of 21.2 months in the need for dialysis. However, the largest randomized placebo-controlled EPPIC trial on patients with moderate to severe CKD^20^ did not report such benefits with AST-120. Nevertheless, a subgroup analysis^21^found that baseline urinary protein to urinary creatinine ratio (UPCR) ≥ 1.0 and hematuria were independent risk factors for ESRD and a reduced eGFR. In summary, while small-scale studies have shown potential renal benefits of AST-120, the largest randomized controlled trial has not yet reached a consensus. Assuming it is beneficial, there is currently no quantitative model available to guide proper decision-making regarding the use of AST-120 in patients. Moreover, the cost of AST-120 usage in Taiwan amounts to 500 US dollars per month. Given the complexity of the issue and the growing need for advanced modeling approaches in CKD care, the application of artificial intelligence (AI) may offer a promising solution^22^.

Based on the above description, this study was initiated to investigate the effect of AST-120 on patient survival using statistical analyses. Upon establishing the impact of AST-120 on patient survival, we further utilized AI to develop improved predictive models for informed decision-making regarding AST-120 usage and its potential impact on patient survival.

## Material and methods

### Study design

Our study was conducted at Taichung Veterans General Hospital (TCVGH) from January 1, 2000, to December 31, 2019, and focused on patients enrolled in our pre-end-stage renal disease (pre-ESRD) program. Notably, our pre-ESRD pay-for-performance (P4P) care program has a strong track record of excellent patient compliance with medication and follow-up periods. The primary objectives of our research were to investigate the impact of AST-120 on patient survival and to develop a predictive model for determining the optimal use of AST-120.

To achieve these objectives, we employed Cox regression analysis, which included both univariate and multivariate analyses, to explore any potential associations between AST-120 usage and patient survival. We also implemented propensity matching to minimize potential confounding factors. The differences in survival between AST-120 users and non-users were assessed through Kaplan–Meier survival curves.

In the event that we identify a significant relationship between patient survival and AST-120 usage, our next step will involve leveraging AI to construct a predictive model with enhanced predictive capabilities. This model aims to assist healthcare professionals in making well-informed decisions regarding the utilization of AST-120 as a treatment option. If the AI model, designed to predict AST-120 usage associated with improved patient survival, outperforms a conventional logistic regression model in terms of predictive power, we will use the Gini index to calculate feature importance.

For enhanced transparency and interpretability of the developed models, we intend to employ SHAP values (Shapley Additive exPlanations). SHAP values will provide insights into the workings of different machine learning models, facilitating a deeper understanding of their predictions and aiding healthcare practitioners in making more informed treatment decisions^23^.

### Definition of target population

In Taiwan, patients with CKD benefit from a comprehensive multidisciplinary care program known as the pre-ESRD P4P program, aimed at enhancing the quality of their healthcare^9^. A significant majority of our CKD patients are actively enrolled in this program. This initiative offers a holistic approach to patient care, with involvement from a diverse group of healthcare professionals^24^. Patients under this program receive thorough assessments and educational support from this dynamic learning healthcare system^9,25^. Notably, our institute stands as one of the prominent healthcare facilities in Taiwan with the highest number of pre-ESRD patients enrolled. By November 2018, we had successfully enrolled over 10,000 CKD patients in this program.

In this study, we included patients who were participants in the pre-ESRD P4P program, based on our in-hospital cohort data spanning from January 1, 2000, to December 31, 2019. To be eligible for inclusion, patients had to meet specific criteria. They were required to be at least 20 years of age and exhibit the following renal function characteristics: Modification of Diet in Renal Disease (MDRD) eGFR less than 45 ml/min/1.73m^2^, as per the classifications outlined by the International Statistical Classification of Diseases and Related Health Problems, 10th Revision (ICD-10) codes N18.3, N18.4, and N18.5, or the ICD-9 code 585.0. Our patient selection process was visually presented in supplementary figure S2. Given the purely analytical nature of this study, the need for informed consent from patients and their family members was waived. The study protocol received thorough review and approval from the Institutional Review Board at TCVGH, bearing approval number CE20026A. All methods employed in this study adhered to the pertinent guidelines and regulations.

It's worth noting that AST-120 usage in Taiwan comes at a monthly cost of 500 US dollars, and it is not covered by the national health insurance. Consequently, not all CKD patients choose to invest in this medication, despite their CKD status. The prescribed dosage of AST-120 consisted of 2 g per package, and the daily amount was determined based on the severity of CKD. At our institute, patients took 2 g once a day if their eGFR ranged between 30–60 ml/min/1.73m^2^. If the eGFR fell within the range of 15–30 ml/min/1.73m^2^, the prescribed dosage was 2 g taken twice a day. For individuals with more severe CKD (eGFR < 15 ml/min/1.73m^2^), the recommended dosage was 2 g taken three times a day. A majority of CKD patients at our institute adhered to these recommendations. Given the high level of compliance observed in our Pre-ESRD P4P program, coupled with consistent reminders from our educators for patients who self-fund AST-120, it is reasonable to assume that all AST-120 users exhibited good compliance with the prescribed regimen.

AST-120, an expensive medication funded by patients themselves, was identified by its Anatomical Therapeutic Chemical (ATC) code (A07BA01), as utilized in our institute. Consequently, patients were classified into four groups based on renal death and AST-120 status. The target population consisted of patients who received AST-120 treatment and maintained renal survival for two years, while the non-target population included all other patients. To identify the potential target population, we employed AI algorithms that analyzed detailed medication records (ATC codes) and medical histories (ICD-9 and ICD-10), as illustrated in supplementary data 2.

### Definition of variables

In our feature engineering and variable selection process, we considered all available variables and potential predictors, encompassing a wide range of factors. These included demographic data such as age and gender, concurrent medications, epidemiological variables, laboratory biomarkers, and comorbidity information. Epidemiological variables covered age (years old), gender, body weight (kg), and body height (cm). Laboratory biomarkers included serum creatinine (mg/dl), eGFR (ml/min/1.73m^2^), daily proteinuria (g/day), urinary albumin creatinine ratio (mg/g), glycated hemoglobin (%), fasting glucose (mg/dl), aspartate aminotransferase (U/L), alanine aminotransferase (U/L), total bilirubin (mg/dl), total cholesterol (mg/dl), high-density lipoprotein (HDL) cholesterol (mg/dl), low-density lipoprotein (LDL) cholesterol (mg/dl), and triglycerides (mg/dl), as well as systolic and diastolic blood pressures (mmHg). Medication history encompassed conditions such as diabetes mellitus, hypertension, hyperlipidemia, gout, congestive heart failure, cerebrovascular disease, cirrhosis, and malignancy. We also collected data related to habits and physical activity, such as walking exercise, brisk walking, running, smoking, and betel nut consumption. Additionally, medication history included the use of erythropoietin, vitamin D, uric acid-lowering agents, angiotensin-converting enzyme inhibitors, angiotensin II receptor blockers, beta-blockers, calcium channel blockers, statins, fibrates, and insulin.

For the sake of broader applicability in future studies, we designated certain variables as necessary features, including age (years old), sex, race, eGFR (ml/min/1.73m^2^), urinary albumin creatinine ratio (mg/g), systolic blood pressure (mmHg), smoking status, diabetes mellitus, and a history of cardiovascular disease (CVD). Other variables were categorized as alternative features, with their inclusion in the deep learning model contingent upon their performance.

### The definition of outcome

The primary outcome of this study focused on patient survival, confirmed by tracking the withdrawal of national health insurance cards. We conducted this study using a right-censoring strategy, allowing us to account for patients who were still alive at the time of data analysis. In addition to survival data, we collected renal function indicators, including serum creatinine and eGFR for further analysis. Patients diagnosed with ESRD were identified as individuals who had undergone dialysis for a minimum duration of 3 months, as evidenced by the acquisition of a certificate of catastrophic illness for dialysis. Furthermore, we analyzed additional surrogate outcomes, including the percentage of patients in stage 5 CKD, time to death, time to ESRD, and time to death or ESRD, to gain a comprehensive understanding of the study's outcomes and implications.

### Model building processes

Two-thirds of the patients were randomly allocated to the training group, while the remaining one-third were assigned to the validation group. In the training group, we developed a predictive algorithm based on deep learning. Subsequently, we validated the generated algorithm using the participants in the validation group. Finally, we compared the outcomes between the AI learning model and the logistic regression model. The target population was identified within the training group. Recognizing the imbalanced sample sizes between the target and non-target populations, we applied different techniques such as up-sampling or the Synthetic Minority Oversampling Technique (SMOTE) to balance these two population samples. We considered both the Deep Neural Network (DNN) and Extreme Gradient Boosting (XGBoost) for the initial deep learning approaches.

To build the deep learning model, we implemented batch normalization to normalize the selected features, setting them as the input layer. The model consisted of three hidden layers, and the output layer was designed with 46 middle layers→46 middle layers→46 middle layers→ one-dimensional output layer. We employed Scaled Exponential Linear Unit (SELU) units in the middle layers and a hard sigmoid unit in the output layer as activation functions.

To prevent overfitting, we introduced dropout layers between the hidden layers. These dropout layers were applied to the outputs of the preceding layer, with a dropout rate set at 0.3. Detailed protocols for this approach were as previously described in the study^26^. We conducted hyperparameter optimization in the training group, with specific parameters optimized for XGBoost and DNN detailed in supplementary table S4. Following model training, we validated the model to assess its performance.

### Model evaluation and comparison

Within the training group, we conducted a comparative analysis of various ROC (Receiver Operating Characteristic) curves, each corresponding to a distinct algorithm. Subsequently, we calculated the AUC (Area Under the ROC Curve) for each algorithm. In addition to the machine learning algorithms, specifically DNN and XGBoost, we compared their outcomes with those derived from the logistic regression model for a comprehensive evaluation of predictive performance.

### Statistical analyses

To assess differences in baseline clinical variables between the target and non-target populations, we employed the independent t-test for continuous variables and the *Chi*-square test for categorical variables. To mitigate potential baseline differences between AST-120 users and non-AST-120 users, we applied propensity score matching. Furthermore, we compared the AUCs using the *Chi*-square test.

The classifier model was developed in the training group. In the validation group, we compared renal and patient survival curves between the target and non-target populations using the Kaplan–Meier method and the Cox-proportional hazard model. We ensured the validity of the Cox’s proportional hazards model by testing the assumption with Scaled Schoenfeld residuals, which were plotted against time (as shown for HospiceReferral in supplementary figure S1), and no violation of the assumption was observed.

All statistical analyses were conducted using SAS for Windows (version 9.4; SAS, Cary, NC). The deep learning algorithms and other machine learning procedures were performed using Keras, TensorFlow 1.10.0, and Python 3.6.5.

### Study approval

This study protocol was reviewed and approved by Institutional Review Board in TCVGH, approval number: CE20026A. all methods were performed in accordance with the relevant guidelines and regulations.

### Consent to participate

The study has been granted an exemption from requiring written informed consent, which was approved by Institutional Review Board in TCVGH (approval number : CE20026A. Chih-Chien Lin, MD, MPH is the Chair, Institutional Review Board (I) in TCVGH and he made the above decision.

## Results

### Revised patient selection for cox model and machine learning analysis

In the initial cohort, a total of 2584 patients were considered. However, we excluded 455 patients due to incomplete questionnaires and medication data, 25 patients due to incomplete laboratory data, and 53 patients due to missing mortality data. As a result, a final dataset comprising 2051 patients was established for the machine learning training dataset (refer to supplementary figure S2). The median follow-up duration was 4.98 years, and the mean follow-up duration was 5.50 years.

### Baseline characteristics data analyzed for association study between using AST-120 or not

Table 1 provides an overview of the baseline characteristics for the AST-120 and non-AST-120 groups. Among the total cohort of 2584 patients, 2199 did not use AST-120, while only 385 took AST-120. There were no statistically significant differences between AST-120 users and non-users in terms of their age, gender, body weight, and body height. However, AST-120 users exhibited a lower baseline eGFR (23.1 ± 14.2 vs. 26 ± 16.9 ml/min/1.73m^2^, *p* = 0.0006), lower total bilirubin levels (0.44 ± 0.3 vs. 0.5 ± 0.6 mg/dl, *p* = 0.0328), a higher prevalence of hyperlipidemia (29.95 vs. 23.18%, *p* = 0.0044), a higher incidence of gout (24.74 vs. 18.18%, *p* = 0.0027), a greater proportion with a family history of kidney disease (7.81 vs. 5.15%, *p* = 0.0359), fewer smokers (30.99 vs. 36.45%, *p* = 0.0398), a higher usage of erythropoietin (EPO) (29.61 vs. 19.83%, *p* < 0.0001), a higher usage of angiotensin II receptor blockers (ARB) (54.55 vs. 44.52%, *p* = 0.0003), a higher usage of calcium channel blockers (36.36 vs. 27.83%, *p* = 0.0092), and a higher usage of statins (36.36 vs. 27.83%, *p* = 0.0008).

**Table 1:** Baseline characteristics according to the usage of AST-120 or not for statistical analysis.

	Overall	AST-120(-)	AST-120( +)	*p* value
Case number	2584	2199	385	
Age (y/o)	65.7 ± 14.5	65.8 ± 14.6	65.1 ± 14	0.4132
Male gender (n, %)	1559 (60.33)	1326 (60.3)	233 (60.52)	0.9353
Body height (cm)	161.8 ± 8.9	161.6 ± 8.9	162.3 ± 8.9	0.2120
Body weight (kg)	64.5 ± 13.4	64.7 ± 13.7	63.7 ± 12.3	0.153
**Laboratory data**				
Serum creatinine (mg/dl)	3.7 ± 2.5	3.7 ± 2.5	3.7 ± 2.4	0.6652
Estimated glomerular filtration rate (eGFR) (ml/min/1.73m^2^)	25.5 ± 16.5	26 ± 16.9	23.1 ± 14.2	**0.0006**
Daily proteinuria (g/day)	2.5 ± 3	2.6 ± 3	2.3 ± 3	0.3748
Urinary albumin creatinine ratio (mg/g)	1281 ± 1428.8	1196.9 ± 1419.3	1566.1 ± 1430.9	0.0181
Log Urinary albumin creatinine ratio (mg/g)	6.1 ± 1.9	5.9 ± 2	6.6 ± 1.6	0.0002
Glycated hemoglobin (HbA1c) (%)	6.8 ± 1.6	6.9 ± 1.6	6.7 ± 1.3	0.1287
Fasting glucose (mg/dl)	120.9 ± 51.7	121.8 ± 53.2	116.6 ± 43.2	0.0633
Aspartate aminotransferase (U/L)	26.6 ± 23.5	26.9 ± 23.8	25.3 ± 21.8	0.3362
Alanine aminotransferase (U/L)	23.4 ± 21.8	23.4 ± 21.6	23.2 ± 23	0.8486
Total bilirubin (mg/dl)	0.5 ± 0.4	0.5 ± 0.4	0.4 ± 0.3	0.0999
Total cholesterol (mg/dl)	184.3 ± 56.2	183.6 ± 54.8	187 ± 61.3	0.3579
High-density lipoprotein (HDL) cholesterol (mg/dl)	49 ± 16.8	49.4 ± 16.9	47.1 ± 16.4	0.1407
Low-density lipoprotein (LDL) cholesterol (mg/dl)	109.9 ± 47.7	109.9 ± 47.1	109.8 ± 50.2	0.9787
Triglyceride (mg/dl)	155.9 ± 106.9	155.6 ± 107.8	156.9 ± 103.5	0.8546
Systolic blood pressure (mmHg)	138.4 ± 22.1	138.6 ± 22.3	137.1 ± 20.8	0.3008
Diastolic blood pressure (mmHg)	76.4 ± 14.3	76.7 ± 14.4	74.9 ± 13.4	0.0624
**Medical history**				
Diabetes mellitus (n, %)	1066 (42.61)	918 (43.34)	148 (38.54)	0.08
Hypertension (n, %)	1797 (71.82)	1517 (71.62)	280 (72.92)	0.6045
Hyperlipidemia (n, %)	606 (24.22)	491 (23.18)	115 (29.95)	**0.0044**
Gout (n, %)	480 (19.18)	385 (18.18)	95 (24.74)	**0.0027**
Congestive heart failure (n, %)	73 (2.92)	57 (2.69)	16 (4.17)	0.1140
Ischemic heart disease (n, %)	93 (3.72)	80 (3.78)	13 (3.39)	0.7089
Cerebrovascular disease (n, %)	94 (3.76)	81 (3.82)	13 (3.39)	0.6773
Liver cirrhosis (n, %)	133 (5.32)	112 (5.29)	21 (5.47)	0.8845
Malignancy (n, %)	179 (7.15)	157 (7.41)	22 (5.73)	0.2389
**Family medical history**				
Diabetes mellitus (n, %)	734 (29.34)	605 (28.56)	129 (33.59)	0.0464
Hypertension (n, %)	824 (32.93)	681 (32.15)	143 (37.24)	0.0510
Heart disease (n, %)	135 (5.4)	112 (5.29)	23 (5.99)	0.5756
Cerebrovascular disease (n, %)	166 (6.63)	138 (6.52)	28 (7.29)	0.5740
Hyperlipidemia (n, %)	34 (1.36)	29 (1.37)	5 (1.3)	0.9167
Kidney disease (n, %)	139 (5.56)	109 (5.15)	30 (7.81)	**0.0359**
Malignancy (n, %)	130 (5.37)	108 (5.31)	22 (5.73)	0.7366
Hereditary disease (n, %)	7 (0.28)	5 (0.24)	2 (0.52)	0.3310
Polycystic kidney disease (n, %)	15 (0.6)	13 (0.61)	2 (0.52)	0.8281
Gout (n, %)	96 (3.84)	80 (3.78)	16 (4.17)	0.7147
**Habit and physical activity**				
Exercise: walking (n, %)	853 (34.09)	712 (33.62)	141 (36.72)	0.2380
Exercise: brisk walking (n, %)	34 (1.36)	26 (1.23)	8 (2.08)	0.1827
Exercise: running (n, %)	35 (1.4)	30 (1.42)	5 (1.3)	0.8607
Smoking (n, %)	891 (35.61)	772 (36.45)	119 (30.99)	**0.0398**
Alcohol drinking (n, %)	632 (25.26)	533 (25.17)	99 (25.78)	0.7983
Betel nut usage	256 (10.23)	222 (10.48)	34 (8.85)	0.333
**Medication history**				
Erythropoiesis-stimulating agents (n, %)	550 (21.28)	436 (19.83)	114 (29.61)	** < 0.0001**
Vitamin D analogue (n, %)	164 (6.35)	133 (6.05)	31 (8.05)	0.1493
Uric acid-lowering agents (n, %)	573 (22.17)	481 (21.87)	92 (23.9)	0.3817
Diuretics (n, %)	1221 (47.25)	1039 (47.25)	182 (47.27)	0.9931
Angiotensin converting enzyme inhibitor (n, %)	220 (8.51)	187 (8.5)	33 (8.57)	0.9651
Angiotensin II receptor blocker (n, %)	1189 (46.01)	979 (44.52)	210 (54.55)	**0.0003**
Beta blocker (n, %)	855 (33.09)	711 (32.33)	144 (37.4)	0.0531
Calcium channel blocker (n, %)	1339 (51.82)	1116 (50.75)	223 (57.92)	**0.0092**
Statin (n, %)	752 (29.1)	612 (27.83)	140 (36.36)	**0.0008**
Fibrate (n, %)	123 (4.76)	105 (4.77)	18 (4.68)	0.9324
Insulin: premix insulin (n, %)	275 (10.64)	239 (10.87)	36 (9.35)	0.3655
Insulin: rapid insulin (n, %)	371 (14.36)	324 (14.73)	47 (12.21)	0.1841
Insulin: basal insulin (n, %)	125 (4.84)	103 (4.68)	22 (5.71)	0.3952
**Outcome**				
Stage 5-CKD (2 years later) (n, %)	712 (32.26)	579 (31.59)	133 (35.56)	0.1341
Mortality (n, %)	885 (34.25)	833 (37.88)	52 (13.51)	** < 0.0001**
End-stage renal disease (ESRD) (n, %)	1351 (52.28)	1181 (53.71)	170 (44.16)	**0.0005**

Significant values are in bold.

Regarding outcomes, AST-120 users exhibited lower mortality (13.51 vs. 37.88%, *p* < 0.0001) and a lower prevalence of ESRD (44.16 vs. 53.17%, *p* = 0.0005). Detailed baseline characteristics among the four groups based on AST-120 users or non-AST-120 users, and their relationship with mortality, can be found in supplementary table S1. Following propensity score matching (1:1 matching in supplementary table S2 and 1:2 matching in supplementary table S3), no significant differences were observed in all variables between AST-120 users and non-AST-120 users, except for all-cause mortality and the need for dialysis.

The results of univariate and multivariate analyses for all-cause mortality are summarized in Table 2. In the univariate analysis, AST-120 usage was associated with a significantly lower all-cause mortality (HR 0.395, 95% CI 0.295–0.522). In the multivariate analysis, AST-120 usage remained significantly associated with lower all-cause mortality (HR 0.41, 95% CI 0.188–0.896). Additionally, age was associated with a higher all-cause mortality (HR 1.055, 95% CI 1.028–1.083). Even after propensity matching, the HRs remained consistently low: 0.444 after 1:1 matching (supplementary table S2), 0.435 after 1:2 matching (supplementary table S3), 0.436 after 1:3 matching, and 0.451 after 1:4 matching.

**Table 2:** Univariate, multivariate analysis, and post-propensity matching analysis for all-cause mortality.

	*p* value	Ratio	95% confidence interval
**Univariate analysis**			
**AST-120 + **	** < 0.0001**	0.395	0.298–0.522
**Multivariate analysis**			
**AST-120 + **	**0.0254**	**0.41**	0.188–0.896
**Age**	** < 0.0001**	**1.055**	1.028–1.083
Male gender	0.9197	0.966	0.491–1.9
Chronic kidney disease stage 2	0.9822	0	0
Chronic kidney disease stage 3	0.7718	0.632	0.028–14.05
Chronic kidney disease stage 4	0.9114	1.207	0.044–33.32
Chronic kidney disease stage 5	0.9484	0.887	0.024–33.12
Urinary albumin creatinine ratio	0.0169	1	1–1
Total bilirubin	0.8704	0.953	0.536–1.694
Estimated glomerular filtration rate	0.6892	1.007	0.972–1.044
Gout	0.4215	1.32	0.671–2.598
Hyperlipidemia	0.4286	1.277	0.697–2.337
Diabetes mellitus	0.9317	1.025	0.587–1.787
Family history of kidney disease	0.0936	0.181	0.024–1.335
Smoking	0.8759	1.058	0.52–2.155
Erythropoiesis-stimulating agents	0.7128	1.154	0.538–2.474
Angiotensin II receptor blocker or angiotensin converting enzyme inhibitor	0.5466	1.203	0.659–2.196
Calcium channel blocker	0.1707	0.617	0.309–1.231
Statin	0.1764	0.663	0.365–1.203
After propensity matching			
**1:1 Matching: AST-1201 + **	** < 0.0001**	**0.444**	0.309–0.636
**1:2Matching: AST-1201 + **	** < 0.0001**	**0.435**	0.310–0.609
**1:3 Matching: AST-1201 + **	** < 0.0001**	**0.436**	0.312–0.609
**1:4 Matching: AST-1201 + **	** < 0.0001**	**0.451**	0.303–0.672

Significant values are in bold.

**Figure 1:**
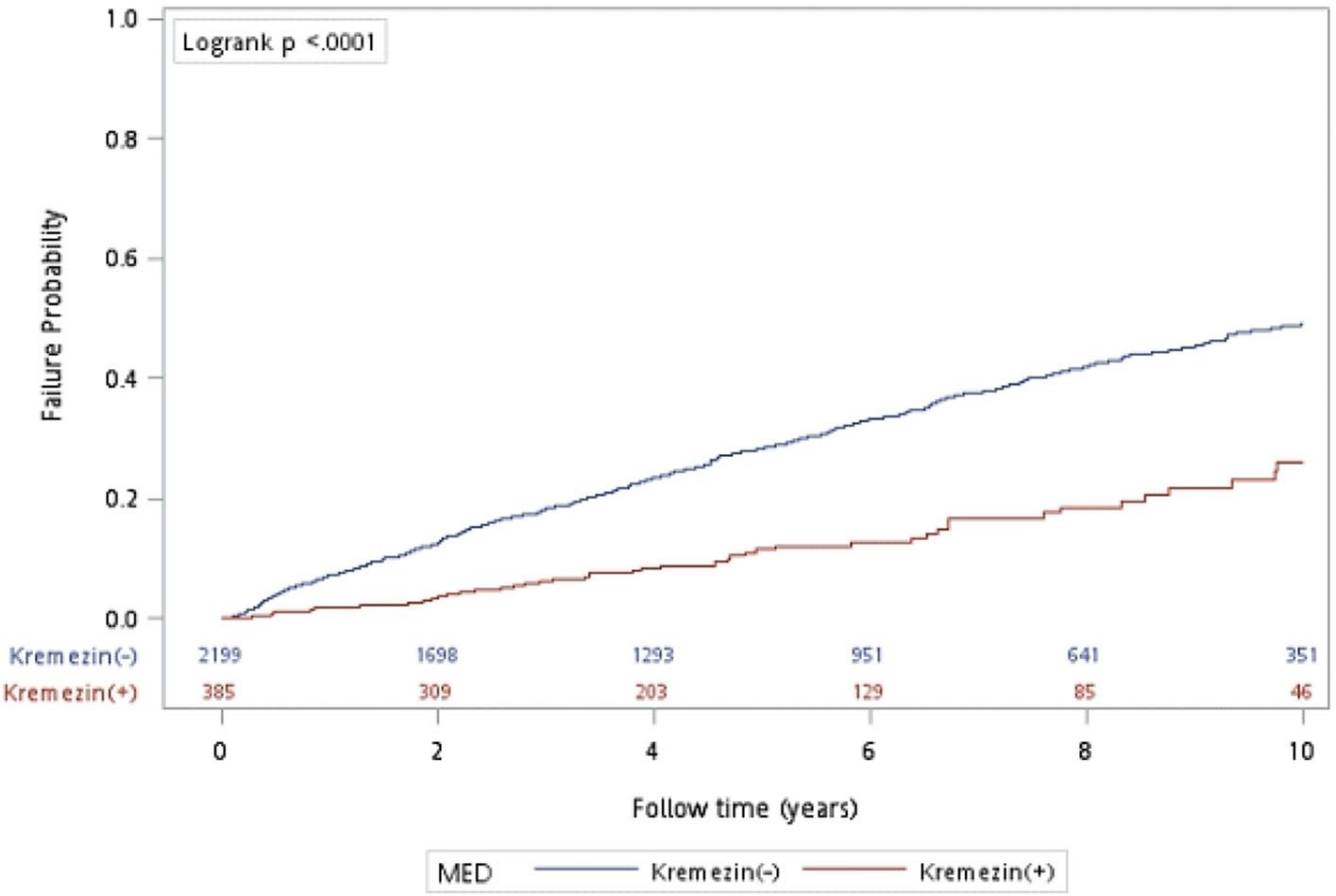
Patient mortality based on using AST-120 or not. Hazard ratio for all-cause mortality = 0.395 (95% CI = 0.298–0.522).

The survival curves for all-cause mortality in the AST-120 and non-AST-120 groups are presented in Fig. 1. Over a 10-year follow-up period, AST-120 users demonstrated significantly lower all-cause mortality compared to non-users (*p* < 0.0001), with a HR of 0.395 (95% CI = 0.295–0.522).

### Baseline characteristics data analyzed with AI

**Figure 2:**
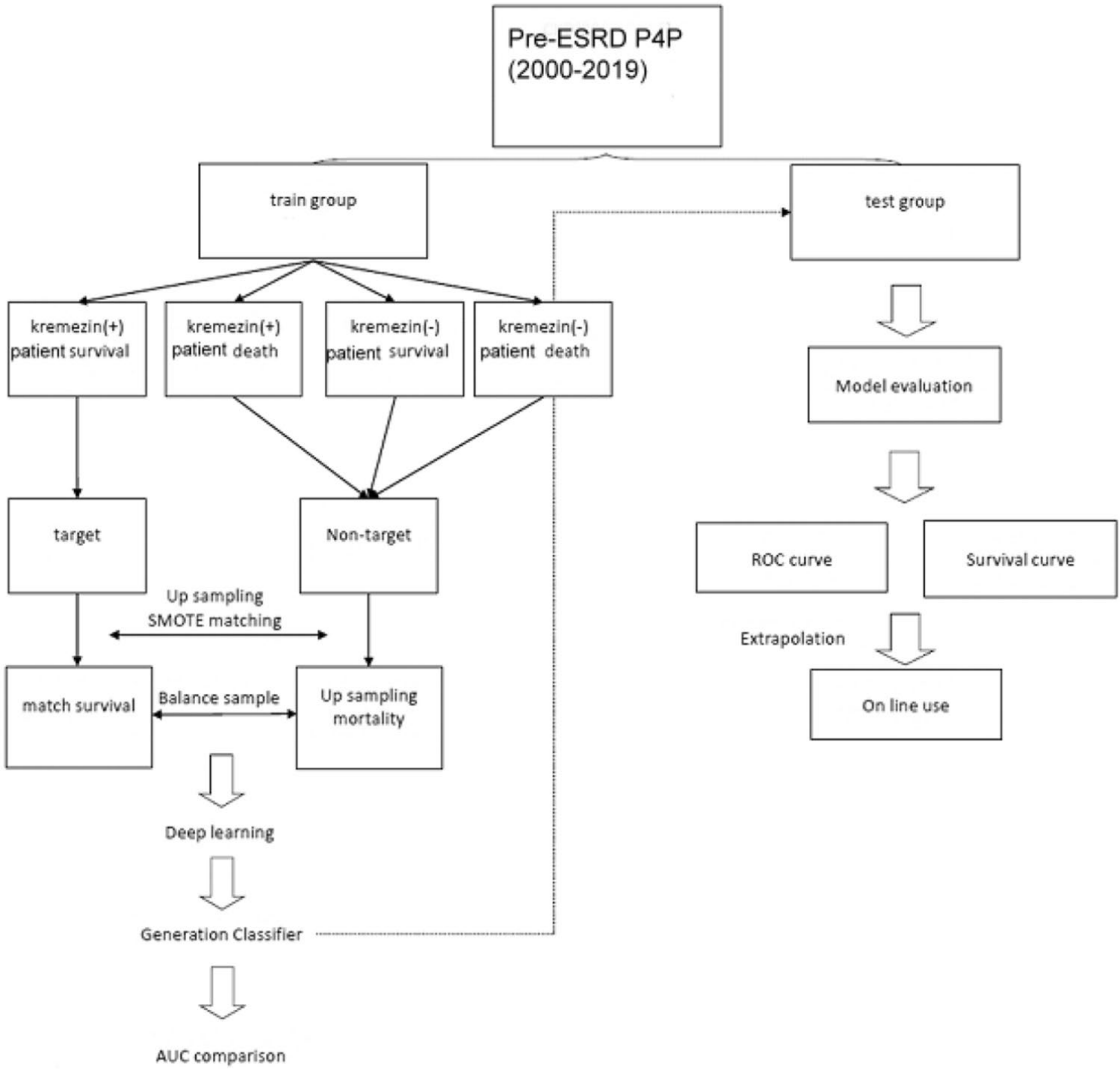
Algorithm for patient’s selection for AI analysis.

The algorithm for patient selection into the training and validation groups is outlined in Fig. 2. We included a total of 2051 CKD patients who were part of our pre-ESR P4P care program. As detailed in Table 3, 1435 patients were allocated to the training group, while 616 patients were designated for the validation group. There were no significant differences between the two groups in terms of age, gender, body weight, body height, laboratory data, medical histories (except for a higher proportion of subjects with hyperlipidemia in the validation group, *p* = 0.0009), family medical histories, medication history, habits and physical activity, and outcomes.

**Table 3:** Baseline characteristics for AI analysis according to train and validation group.

	Overall	Train	Validation	p-value
Case number	2051	1435	616	
Age (y/o)	65.6 ± 14.5	65.9 ± 14.4	65 ± 14.6	0.199
Male gender (n, %)	1236 (60.26)	868 (60.49)	368 (59.74)	0.7511
Body height (cm)	161.8 ± 8.9	161.9 ± 8.9	161.7 ± 9	0.7240
Body weight (kg)	64.7 ± 13.5	64.7 ± 13.5	64.7 ± 13.5	0.9839
**Laboratory data**				
Serum creatinine (mg/dl)	3.6 ± 2.5	3.6 ± 2.5	3.7 ± 2.6	0.3610
Estimated glomerular filtration rate (eGFR) (ml/min/1.73m^2^)	24.8 ± 15.4	24.9 ± 15.2	24.8 ± 15.9	0.9456
Daily proteinuria (g/day)	2.6 ± 3.1	2.6 ± 3.2	2.6 ± 3	0.9660
Urinary albumin creatinine ratio (mg/g)	1286.4 ± 1440.2	1319 ± 1447.7	1194.7 ± 1420.6	0.4154
Log Urinary albumin creatinine ratio (mg/g)	6.1 ± 1.9	6.1 ± 1.9	6 ± 1.9	0.6285
Glycated hemoglobin (HbA1c) (%)	6.8 ± 1.6	6.8 ± 1.6	6.8 ± 1.5	0.3779
Fasting glucose (mg/dl)	120.8 ± 52.3	122.2 ± 56.1	117.7 ± 41.9	0.0667
Aspartate aminotransferase (U/L)	26.9 ± 24.2	27.2 ± 26.4	26.1 ± 17.7	0.4085
Alanine aminotransferase (U/L)	23.6 ± 22.4	23.4 ± 22.6	24.1 ± 21.9	0.5549
Total bilirubin (mg/dl)	0.4 ± 0.4	0.5 ± 0.4	0.4 ± 0.3	0.2340
Total cholesterol (mg/dl)	183.9 ± 56.8	184.3 ± 57.6	183.1 ± 55	0.712
High-density lipoprotein (HDL) cholesterol (mg/dl)	48.8 ± 16.7	49 ± 16.9	48.4 ± 16.3	0.6861
Low-density lipoprotein (LDL) cholesterol (mg/dl)	109.4 ± 47.5	108.2 ± 48.2	112.1 ± 46.1	0.1757
Triglyceride (mg/dl)	155.7 ± 107	154.4 ± 104.1	158.5 ± 113.3	0.5177
Systolic blood pressure (mmHg)	138.3 ± 22.2	138.5 ± 22.5	137.8 ± 21.4	0.5501
Diastolic blood pressure (mmHg)	76.4 ± 14.3	76.6 ± 14.3	75.9 ± 14.2	0.3648
**Medical history**				
Diabetes mellitus (n, %)	881 (42.95)	628 (43.76)	253 (41.07)	0.259
Hypertension (n, %)	1491 (72.7)	1045 (72.82)	446 (72.4)	0.8449
Gout (n, %)	412 (20.09)	293 (20.42)	119 (19.32)	0.5687
Congestive heart failure (n, %)	62 (3.02)	36 (2.51)	26 (4.22)	0.0379
Ischemic heart disease (n, %)	83 (4.05)	61 (4.25)	22 (3.57)	0.4741
Cerebrovascular disease (n, %)	78 (3.8)	48 (3.34)	30 (4.87)	0.0978
Liver cirrhosis (n, %)	121 (5.9)	88 (6.13)	33 (5.36)	0.4946
Malignancy (n, %)	153 (7.46)	114 (7.94)	39 (6.33)	0.2025
Hyperlipidemia (n, %)	526 (25.65)	338 (23.55)	188 (30.52)	**0.0009**
**Family medical history**				
Diabetes mellitus (n, %)	643 (31.35)	441 (30.73)	202 (32.79)	0.3565
Hypertension (n, %)	728 (35.49)	517 (36.03)	211 (34.25)	0.4413
Heart disease (n, %)	120 (5.85)	88 (6.13)	32 (5.19)	0.4069
Cerebrovascular disease (n, %)	136 (6.63)	94 (6.55)	42 (6.82)	0.8233
Hyperlipidemia (n, %)	30 (1.46)	25 (1.74)	5 (0.81)	0.1076
Kidney disease (n, %)	122 (5.95)	76 (5.3)	46 (7.47)	0.0567
Malignancy (n, %)	112 (5.46)	73 (5.09)	39 (6.33)	0.2557
Hereditary disease (n, %)	5 (0.24)	3 (0.21)	2 (0.32)	0.6265
Polycystic kidney disease (n, %)	12 (0.59)	7 (0.49)	5 (0.81)	0.378
Gout (n, %)	79 (3.85)	59 (4.11)	20 (3.25)	0.3509
**Habit and physical activity**				
Exercise: walking (n, %)	710 (34.62)	484 (33.73)	226 (36.69)	0.1965
Exercise: brisk walking (n, %)	31 (1.51)	22 (1.53)	9 (1.46)	0.9024
Exercise: running (n, %)	29 (1.41)	18 (1.25)	11 (1.79)	0.3501
Smoking (n, %)	740 (36.08)	527 (36.72)	213 (34.58)	0.3534
Alcohol drinking (n, %)	530 (25.84)	384 (26.76)	146 (23.7)	0.147
Betel nut usage	234 (11.41)	158 (11.01)	76 (12.34)	0.3861
**Medication history**				
Erythropoiesis-stimulating agents (n, %)	499 (24.33)	344 (23.97)	155 (25.16)	0.5647
Vitamin D analogue (n, %)	149 (7.26)	98 (6.83)	51 (8.28)	0.2462
Uric acid-lowering agents (n, %)	511 (24.91)	364 (25.37)	147 (23.86)	0.4709
Diuretics (n, %)	1104 (53.83)	776 (54.08)	328 (53.25)	0.7296
Angiotensin converting enzyme inhibitor (n, %)	181 (8.82)	117 (8.15)	64 (10.39)	0.1017
Angiotensin II receptor blocker (n, %)	1088 (53.05)	746 (51.99)	342 (55.52)	0.1416
Beta blocker (n, %)	783 (38.18)	540 (37.63)	243 (39.45)	0.4374
Calcium channel blocker (n, %)	1208 (58.9)	843 (58.75)	365 (59.25)	0.8304
Statin (n, %)	696 (33.93)	482 (33.59)	214 (34.74)	0.6137
Fibrate (n, %)	104 (5.07)	79 (5.51)	25 (4.06)	0.171
Insulin: premix insulin (n, %)	252 (12.29)	182 (12.68)	70 (11.36)	0.4041
Insulin: rapid insulin (n, %)	351 (17.11)	249 (17.35)	102 (16.56)	0.6618
Insulin: basal insulin (n, %)	122 (5.95)	83 (5.78)	39 (6.33)	0.631
AST-120 (n, %)	363 (17.7)	262 (18.26)	101 (16.40)	0.3112
**Outcome**				
ESRD (n, %)	660 (32.18)	460 (32.06)	200 (32.47)	0.8548
Mortality (n, %)	604 (29.45)	423 (29.48)	181 (29.38)	0.9658
Mortality or ESRD	1044 (50.9)	728 (50.73)	316 (51.3)	0.8139
Time to death	4.9 ± 3.5	4.8 ± 3.5	5 ± 3.4	0.3559
Time to ESRD	4.2 ± 4	4.2 ± 4	4.2 ± 3.9	0.3559
Time to death or ESRD	3.4 ± 3.7	3.4 ± 3.7	3.4 ± 3.7	0.6255

Significant values are in bold.

The mean age of participants with moderate CKD (mean eGFR of 24.8 ± 15.4 ml/min/1.73m^2^) was 65.6 ± 14.5 years. Over 40% of patients had diabetes mellitus (42.95%), and more than 70% had hypertension (71.2%). A majority of patients were on renin–angiotensin system inhibitors (61.87%), including 8.82% using angiotensin-converting enzyme inhibitors and 53.05% using angiotensin II receptor blockers. In this cohort, only 17.7% of patients were taking AST-120. During the follow-up period, nearly 30% (29.45%) of patients passed away, and 32.18% of patients progressed to ESRD. The average time to death was 4.9 ± 3.5 years, and the time to ESRD was 4.2 ± 4 years.

### Predictive power of models for the decision of AST-120 or not by DNN, XGBoost and logistic regression test

**Figure 3:**
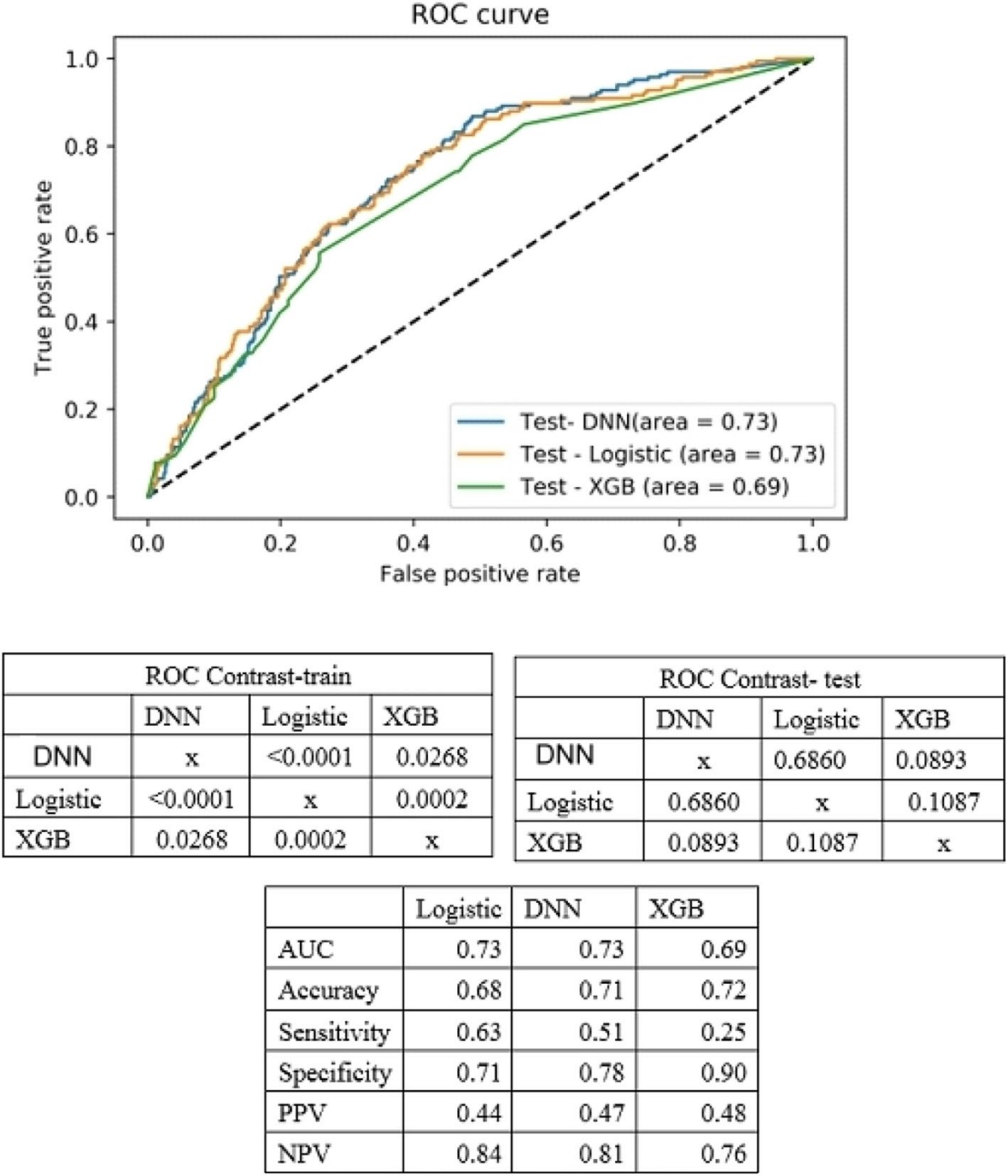
ROC curve for DNN, XGBoost and logistic regression test.

Figure 3 displays the ROC curves for DNN, XGBoost, and logistic regression tests. In the training group, statistical significance (*p* < 0.05) was observed when comparing the AUCs in DNN, logistic regression, and XGBoost. However, in the test group, there were no significant differences in AUCs among DNN, logistic regression, and XGBoost.

Regarding accuracy, specificity, and positive predictive value, the best-performing model was XGBoost (with values of 0.72, 0.90, and 0.48, respectively). In terms of sensitivity and negative predictive value, logistic regression had the highest values (at 0.63 and 0.84, respectively). The AUC values were 0.73 for logistic regression, 0.73 for DNN, and 0.69 for XGBoost, indicating similar predictive powers for the decision of whether to use AST-120 or not.

## Discussion

AST-120 usage was associated with a reduced incidence of ESRD (44.16% vs. 53.71%, *p* = 0.0005) and lower mortality (13.51% vs. 37.88%, *p* < 0.0001). The use of AST-120 was independently linked to a significant reduction in all-cause mortality, with a 59% risk reduction. This effect remained consistent even after adjusting for factors such as age, gender, CKD stage, urinary albumin creatinine ratio, eGFR, gout, hyperlipidemia, diabetes mellitus, smoking, erythropoietin, ARB or ACEi use, calcium channel blocker, and statin use.

These findings suggest that the impact of AST-120 on a patient's survival operates through mechanisms beyond atherosclerosis or metabolic syndrome. Previous studies have reported the association between serum IS levels and patient mortality. In a smaller-scale study involving 150 participants, the highest IS tertile was associated with significantly higher all-cause and cardiovascular mortality (*p* = 0.001 and 0.012, respectively)^13^. The predictive power of IS for all-cause mortality was independent of age, gender, diabetes mellitus, albumin, hemoglobin, phosphate, and aortic calcification^13^. In another study with 147 subjects, IS levels were associated with major adverse cardiovascular events, with an AUC of 0.708. These findings suggest that IS may play a critical role in predicting cardiovascular disease in CKD patients^27^. A meta-analysis involving 1572 CKD patients, which included 10 prospective and one cross-sectional study^28^ found that PC concentration was significantly associated with all-cause mortality (pooled odds ratio 1.16, 95% confidence interval 1.03 to 1.30, *p* = 0.013). Elevated IS levels were also significantly associated with a higher risk of all-cause mortality (pooled odds ratio 1.10, 95% confidence interval 1.03 to 1.17, *p* = 0.003). Elevated PC levels were significantly associated with a higher risk of cardiovascular disease (pooled odds ratio 1.28, 95% confidence interval 1.10 to 1.50, *p* = 0.002)^28^. In summary, serum IS and PC levels have been linked to poorer survival in CKD patients. Notably, the previous observational studies and the meta-analysis had limited case numbers, such as the meta-analysis with only 1572 patients. Moreover, they did not observe a direct association between the intervention involving IS and PC (the use of AST-120) and mortality, unlike the relationship between serum IS/PC levels and mortality. Our present study, with a larger number of cases (1584 CKD patients), has established a direct association between AST-120 usage and patient mortality.

The lack of consensus regarding the association between AST-120 and all-cause mortality can be attributed to inadequate dosing and poor medication compliance. AST-120 has been shown to reduce serum IS levels in a dose-dependent manner, and the effect on patient survival is also linked to the dosage of AST-120^29^. A post-hoc subgroup analysis of randomized controlled trials conducted in the USA^30^ revealed a significant difference between treatment groups in achieving the primary endpoint (HR 0.74; 95%CI 0.56–0.97) in the population with a medication compliance rate of ≥ 67%. However, drug adherence to AST-120 is generally as low as 70% in Japan^31^. Efforts have been made to enhance AST-120's compliance, such as changing its formulation into fine granules that quickly disintegrate with a small amount of water without spreading inside the mouth. This new formulation of AST-120 is expected to improve the ease of taking the medication and promote better adherence to treatment. Comparatively, AST-120 tablets have demonstrated good palatability and can increase medication adherence when compared to AST-120 fine granules, with 70% of patients preferring the switch to tablets^32^.

CVD plays a significant role in the overall mortality of CKD patients, and CKD is often considered as being equivalent to coronary heart disease^33^. As a result, the impact of AST-120 on all-cause mortality primarily stems from its ability to reduce CVD. However, the exact mechanism by which AST-120, through the reduction of IS and PC, affects a patient's mortality remains not fully understood. IS is known to enhance the hypermethylation of Klotho, which can contribute to vascular calcification in CKD^34^. In hypertensive rat models, IS has been shown to promote aortic calcification by inducing the expression of osteoblast-specific proteins^35^. It also promotes cell senescence along with aortic calcification and the expression of senescence-related proteins^36^. Exposure to IS and PC has been found to activate inflammation and coagulation signaling pathways in the aorta, which are causally implicated in toxin-induced arterial calcification^37^. Moreover, a retrospective analysis involving 199 CKD patients revealed that the aortic calcification index was significantly lower in patients who took AST-120 [12.2% (2.5–30.3%) vs. 25.7% (13.4–45.3%), *p* < 0.001]^38^.

Deep learning, a machine learning approach inspired by the functioning of the human brain, is characterized by the combination of layered artificial neurons^39^. It has shown great promise in various clinical scenarios, especially when there is an abundance of data but limited expertise in the specific domain. However, in our study, we encountered challenges in developing a more effective model to predict the impact of AST-120 on mortality in CKD patients (with an AUC of 0.73 in DNN and logistic regression, and 0.69 in XGBoost). Several factors could explain these outcomes. First, deep learning algorithms require extensive training datasets, and their advantage lies in the volume of data available^40^. When provided with sufficient data, deep learning models tend to outperform shallow neural networks, traditional machine learning methods, and basic statistical analyses. In our study, the number of AST-120 users was limited to 385, while non-AST-120 users numbered 2199. These data volumes fell below the threshold necessary to showcase the superiority of DNN. Second, the dataset used for deep learning should ideally be comprehensive, unbiased, and of high quality. Moreover, a longer follow-up period is needed to generate new data that could enhance model performance. Third, it is possible that our AI models lacked some key features. Important variables may have been missing from the study, such as information about protein diet (including the ratio of animal protein intake and adherence to low or very low protein diets), the specific dosage of AST-120, patients' compliance with AST-120, and details about the causes of CKD and other acute kidney injuries. The inclusion of additional variables or critical features could lead to more accurate predictions, but to leverage the full potential of deep learning, larger datasets would be required. Deep learning models with larger architectures are especially data-intensive and tend to perform better with an expanded feature set. Our study may have suffered from a shortage of relevant variables for analysis, which could have limited the model's performance.

Our study is subject to several limitations. Firstly, we did not document the specific dosage of AST-120 administered to patients, although the majority of CKD patients at our institution typically follow recommendations of 2 g/day in stage 3 CKD, 4 g/day in stage 4 CKD, and 6 g/day in stage 5 CKD. Secondly, we lacked data on the adherence of patients to AST-120. However, it's worth noting that patients in pre-ESRD P4P care programs generally display good compliance, and they receive consistent reminders from our educators, particularly those who self-pay for AST-120. Additionally, we did not have data on serum IS and PC levels to confirm the impact of AST-120 on these specific biomarkers. Thirdly, our study did not include detailed information on the specific causes of mortality. Fourthly, due to our study design, we cannot establish a causal relationship between AST-120 and all-cause mortality. In future studies, we plan to collect data from a larger sample of patients, including information on the specific dosage, compliance, and duration of AST-120 usage. Fifthly, there is the presence of selection bias, as more affluent individuals are more likely to afford and access AST-120 treatment. Moreover, physicians who prescribe AST-120 for their patients may be more experienced or have a more proactive approach to therapy. These factors cannot be addressed through propensity score matching. Lastly, it's important to note that our study is retrospective and non-randomized, which implies that there may still be unknown confounding factors that influence our results.

## Conclusion

In this cohort study involving CKD patients, AST-120 usage was associated with a decrease in patient mortality. Nevertheless, our artificial intelligence model, as implemented with the existing architecture and algorithm using our dataset, did not demonstrate superior performance when compared to traditional statistical analysis in predicting the decision to prescribe AST-120 or not.

### Supplementary Information

Supplementary Information.

## Data Availability

All relevant data are within the paper and its Supporting Information files.

## References

[1] K/DOQI clinical practice guidelines for chronic kidney disease (2002). evaluation, classification, and stratification. Am. J. Kidney Dis..

[2] Levey AS, Stevens LA, Coresh J (2009). Conceptual model of CKD: Applications and implications. Am. J. Kidney Dis..

[3] Bikbov B, Purcell CA, Levey AS, Smith M, Abdoli A, Abebe M, Owolabi MO (2020). Global, regional, and national burden of chronic kidney disease, 1990–2017: a systematic analysis for the Global Burden of Disease Study 2017. Lancet (Lond. Engl.).

[4] Carney EF (2020). The impact of chronic kidney disease on global health. Nat. Rev. Nephrol..

[5] Wen CP (2008). All-cause mortality attributable to chronic kidney disease: a prospective cohort study based on 462 293 adults in Taiwan. Lancet (Lond. Engl.).

[6] Jha V, Wang AY, Wang H (2012). The impact of CKD identification in large countries: the burden of illness. Nephrol. Dial. Transpl..

[7] Garneata L, Stancu A, Dragomir D, Stefan G, Mircescu G (2016). Ketoanalogue-supplemented vegetarian very low-protein diet and CKD progression. J. Am. Soc. Nephrol..

[8] Yen CL (2018). Does a supplemental low-protein diet decrease mortality and adverse events after commencing dialysis? A nationwide cohort study. Nutrients.

[9] Hsieh HM (2017). Economic evaluation of a pre-ESRD pay-for-performance programme in advanced chronic kidney disease patients. Nephrol. Dial. Transpl..

[10] Vanholder RC, Glorieux GL (2003). An overview of uremic toxicity. Hemodial. Int. Int. Symp. Home Hemodial..

[11] Meyer TW, Hostetter TH (2012). Uremic solutes from colon microbes. Kidney Int..

[12] Gryp T, Vanholder R, Vaneechoutte M, Glorieux G (2017). p-Cresyl Sulfate. Toxins.

[13] Barreto FC (2009). Serum indoxyl sulfate is associated with vascular disease and mortality in chronic kidney disease patients. Clin. J. Am. Soc. Nephrol. CJASN.

[14] Miyazaki T, Ise M, Seo H, Niwa T (1997). Indoxyl sulfate increases the gene expressions of TGF-beta 1, TIMP-1 and pro-alpha 1(I) collagen in uremic rat kidneys. Kidney Int. Suppl..

[15] Bammens B, Evenepoel P, Keuleers H, Verbeke K, Vanrenterghem Y (2006). Free serum concentrations of the protein-bound retention solute p-cresol predict mortality in hemodialysis patients. Kidney Int..

[16] Shafi T (2015). Free levels of selected organic solutes and cardiovascular morbidity and mortality in hemodialysis patients: Results from the retained organic solutes and clinical outcomes (ROSCO) investigators. PLoS ONE.

[17] Hatakeyama S (2012). Effect of an oral adsorbent, AST-120, on dialysis initiation and survival in patients with chronic kidney disease. Int. J. Nephrol..

[18] Akizawa T (2009). Effect of a carbonaceous oral adsorbent on the progression of CKD: a multicenter, randomized, controlled trial. Am. J. Kidney Dis..

[19] Maeda K (2009). Long-term effects of the oral adsorbent, AST-120, in patients with chronic renal failure. J. Int. Med. Res..

[20] Schulman G (2015). Randomized placebo-controlled EPPIC trials of AST-120 in CKD. J. Am. Soc. Nephrol. JASN.

[21] Schulman G (2018). Risk factors for progression of chronic kidney disease in the EPPIC trials and the effect of AST-120. Clin. Exp. Nephrol..

[22] Davenport T, Kalakota R (2019). The potential for artificial intelligence in healthcare. Fut. Healthcare J..

[23] Lundberg, S. M. & Lee, S.-I. A unified approach to interpreting model predictions. *Adv. Neural Inf. Process. Syst.* **30** (2017).

[24] Lin MY (2018). Effect of national pre-ESRD care program on expenditures and mortality in incident dialysis patients: A population-based study. PloS One.

[25] Wu MY, Wu MS (2018). Taiwan renal care system: A learning health-care system. Nephrology (Carlton).

[26] Khan SH, Hayat M, Porikli F (2019). Regularization of deep neural networks with spectral dropout. Neural Netw..

[27] Fan PC (2019). Serum indoxyl sulfate predicts adverse cardiovascular events in patients with chronic kidney disease. J. Formos Med. Assoc..

[28] Lin CJ, Wu V, Wu PC, Wu CJ (2015). Meta-analysis of the associations of p-Cresyl Sulfate (PCS) and Indoxyl Sulfate (IS) with cardiovascular events and all-cause mortality in patients with chronic renal failure. PloS one.

[29] Schulman G (2006). A multicenter, randomized, double-blind, placebo-controlled, dose-ranging study of AST-120 (Kremezin) in patients with moderate to severe CKD. Am. J. Kidney Dis..

[30] Schulman G (2016). The effects of AST-120 on chronic kidney disease progression in the United States of America: a post hoc subgroup analysis of randomized controlled trials. BMC Nephrol..

[31] Tomino Y (2018). Importance of AST-120 (Kremezin®) adherence in a chronic kidney disease patient with diabetes. Case Rep. Nephrol. Dial..

[32] Riku Setogawa, S. U., Yasuharu Kashiwagura, Shimako Tanaka, and Noriyuki Namiki. Difference in Palatability Among the Formulations of Kremezin®, an Oral Absorbent of Uremic Toxins, in Chronic Kidney Disease (CKD) Patients. *Meeting of Advancing Pharmaceutical Sciences* (2019).

[33] Sarnak MJ (2003). Kidney disease as a risk factor for development of cardiovascular disease: a statement from the American Heart Association Councils on Kidney in Cardiovascular Disease, High Blood Pressure Research, Clinical Cardiology, and Epidemiology and Prevention. Circulation.

[34] Chen J (2016). Indoxyl sulfate enhance the hypermethylation of klotho and promote the process of vascular calcification in chronic kidney disease. Int J Biol Sci.

[35] Adijiang A, Goto S, Uramoto S, Nishijima F, Niwa T (2008). Indoxyl sulphate promotes aortic calcification with expression of osteoblast-specific proteins in hypertensive rats. Nephrol. Dial. Transpl..

[36] Adijiang A, Higuchi Y, Nishijima F, Shimizu H, Niwa T (2010). Indoxyl sulfate, a uremic toxin, promotes cell senescence in aorta of hypertensive rats. Biochem. Biophys. Res. Commun..

[37] Opdebeeck B (2019). Indoxyl sulfate and p-cresyl sulfate promote vascular calcification and associate with glucose intolerance. J. Am. Soc. Nephrol. JASN.

[38] Goto S (2013). Association between AST-120 and abdominal aortic calcification in predialysis patients with chronic kidney disease. Clin. Exp. Nephrol..

[39] Kalmet PHS (2020). Deep learning in fracture detection: a narrative review. Acta Orthopaedica.

[40] Guo Q, Jin S, Li M, Yang Q, Xu K, Ju Y, Liu Y (2020). Application of deep learning in ecological resource research: Theories, methods, and challenges. Sci. China Earth Sci..

