# Supplementary material for: Effects of AST-120 on mortality in patients with chronic kidney disease modeled by artificial intelligence or traditional statistical analysis
Source: Sci Rep. 2024 Jan 6;14:738. doi: 10.1038/s41598-024-51498-6 (PMC10771424; doi:10.1038/s41598-024-51498-6)

Supplementary data

Table S1. Baseline characteristics among four groups: Kremeizn and mortality or not.

|  | Overall | AST-120(-) death(-) | AST-120(+) death(-) | AST-120(-) death(+) | AST-120(+) death(+) | P value |
| --- | --- | --- | --- | --- | --- | --- |
| Case number | 2584 | 1366 | 333 | 833 | 52 |  |
| Age (y/o) | 65.7±14.5 | 62.1±14.5 | 63.5±13.4 | 71.7±12.7 | 75.3±12.9 | <.0001 |
| Male gender (n, %) | 1559(60.33) | 787(57.61) | 201(60.36) | 539(64.71) | 32(61.54) | 0.0119 |
| Body height (cm) | 168.5±323.5 | 174.4±434.8 | 162.5±9 | 160.5±9.7 | 158.5±17.6 | 0.8192 |
| Body weight (kg) | 64.5±13.4 | 65.7±13.7 | 64±12.2 | 62.5±13.3 | 61.7±12.3 | <.0001 |
| **Laboratory data** |  |  |  |  |  |  |
| Serum creatinine (mg/dl) | 6.5±14.3 | 6.9±15.5 | 5.4±10.4 | 6.3±13.3 | 8.1±14.2 | 0.2941 |
| Estimated glomerular filtration rate (eGFR) | 24.4±15.5 | 26.5±16.5 | 23.9±15 | 21.2±13.3 | 23.8±11.8 | <.0001 |
| Daily proteinuria (g/day) | 2.9±5.2 | 2.6±3.4 | 3.4±9.9 | 3.1±3.8 | 3.3±4.3 | 0.2452 |
| Urinary albumin creatinine ratio (mg/g) | 1722.3±2283 | 1433.4±1919.7 | 2236.4±2586.9 | 1892.7±2587 | 2245.2±3182.9 | 0.0108 |
| Glycated hemoglobin (HbA1c) (%) | 6.8±1.6 | 6.8±1.6 | 6.7±1.4 | 7.1±1.7 | 6.7±1.1 | 0.0201 |
| Fasting glucose (mg/dl) | 120.9±51.7 | 119.3±46.2 | 116.5±45.1 | 126±63.3 | 117±28.8 | 0.0336 |
| Aspartate aminotransferase (U/L) | 27.8±38.2 | 25.8±29.1 | 25.5±23.1 | 32.7±55.1 | 23.3±9.1 | 0.026 |
| Alanine aminotransferase (U/L) | 24±28.3 | 23.5±26.7 | 23.5±24 | 25.2±33.5 | 20.5±13 | 0.5868 |
| Total bilirubin (mg/dl) | 0.5±0.5 | 0.5±0.5 | 0.4±0.3 | 0.5±0.7 | 0.5±0.3 | 0.3849 |
| Total cholesterol (mg/dl) | 184.3±56.2 | 185.7±55.8 | 186.6±59.4 | 179.1±52.5 | 189.6±73.6 | 0.1804 |
| High-density lipoprotein (HDL) cholesterol (mg/dl) | 49±16.8 | 49.4±16.7 | 46.8±15.3 | 49.4±17.2 | 49.8±24.1 | 0.4515 |
| low-density lipoprotein (LDL) cholesterol (mg/dl) | 109.9±47.7 | 110.5±47.2 | 111.4±50.4 | 108.2±46.9 | 96.9±47.2 | 0.3639 |
| Triglyceride (mg/dl) | 161.7±152.3 | 163.7±156.9 | 171.1±183.1 | 150.3±108.5 | 140.7±101.3 | 0.2974 |
| Systolic blood pressure (mmHg) | 138.4±22.1 | 138.9±22.2 | 137.5±20.7 | 137.9±22.7 | 134.1±21.5 | 0.4845 |
| Diastolic blood pressure (mmHg) | 76.4±14.3 | 77.3±14.3 | 75.5±13.4 | 75.2±14.6 | 71±12.9 | 0.0034 |
| **Medical history** |  |  |  |  |  |  |
| Diabetes mellitus (n, %) | 1066(42.61) | 514(38.24) | 129(38.86) | 404(52.2) | 19(36.54) | <.0001 |
| Hypertension (n, %) | 1797(71.82) | 950(70.68) | 240(72.29) | 567(73.26) | 40(76.92) | 0.503 |
| Hyperlipidemia (n, %) | 606(24.22) | 331(24.63) | 98(29.52) | 160(20.67) | 17(32.69) | 0.0057 |
| Gout (n, %) | 480(19.18) | 252(18.75) | 83(25) | 133(17.18) | 12(23.08) | 0.0193 |
| Congestive heart failure (n, %) | 73(2.92) | 35(2.6) | 12(3.61) | 22(2.84) | 4(7.69) | 0.1553 |
| Ischemic heart disease (n, %) | 93(3.72) | 34(2.53) | 9(2.71) | 46(5.94) | 4(7.69) | 0.0002 |
| Cerebrovascular disease (n, %) | 94(3.76) | 39(2.9) | 11(3.31) | 42(5.43) | 2(3.85) | 0.0311 |
| Liver cirrhosis (n, %) | 133(5.32) | 64(4.76) | 19(5.72) | 48(6.2) | 2(3.85) | 0.5015 |
| Malignancy (n, %) | 179(7.15) | 82(6.1) | 16(4.82) | 75(9.69) | 6(11.54) | 0.003 |
| **Family medical history** |  |  |  |  |  |  |
| Diabetes mellitus (n, %) | 734(29.34) | 432(32.14) | 115(34.64) | 173(22.35) | 14(26.92) | <.0001 |
| Hypertension (n, %) | 824(32.93) | 505(37.57) | 127(38.25) | 176(22.74) | 16(30.77) | <.0001 |
| Heart disease (n, %) | 135(5.4) | 75(5.58) | 18(5.42) | 37(4.78) | 5(9.62) | 0.4792 |
| Cerebrovascular disease (n, %) | 166(6.63) | 93(6.92) | 25(7.53) | 45(5.81) | 3(5.77) | 0.6799 |
| Hyperlipidemia (n, %) | 34(1.36) | 24(1.79) | 3(0.9) | 5(0.65) | 2(3.85) | 0.0532 |
| Kidney disease (n, %) | 139(5.56) | 84(6.25) | 26(7.83) | 25(3.23) | 4(7.69) | 0.0048 |
| Malignancy (n, %) | 130(5.37) | 79(5.99) | 20(6.02) | 29(4.04) | 2(3.85) | 0.2612 |
| Hereditary disease (n, %) | 7(0.28) | 3(0.22) | 2(0.6) | 2(0.26) | 0(0) | 0.6705 |
| Polycystic kidney disease (n, %) | 15(0.6) | 11(0.82) | 1(0.3) | 2(0.26) | 1(1.92) | 0.2021 |
| Gout (n, %) | 96(3.84) | 61(4.54) | 14(4.22) | 19(2.45) | 2(3.85) | 0.115 |
| **Habit and physical activity** |  |  |  |  |  |  |
| Exercise: walking (n, %) | 853(34.09) | 469(34.9) | 121(36.45) | 243(31.4) | 20(38.46) | 0.2455 |
| Exercise: brisk walking (n, %) | 34(1.36) | 24(1.79) | 8(2.41) | 2(0.26) | 0(0) | 0.0065 |
| Exercise: running (n, %) | 35(1.4) | 26(1.93) | 5(1.51) | 4(0.52) | 0(0) | 0.0475 |
| Smoking (n, %) | 891(35.61) | 449(33.41) | 97(29.22) | 323(41.73) | 22(42.31) | <.0001 |
| Alcohol drinking (n, %) | 632(25.26) | 317(23.59) | 85(25.6) | 216(27.91) | 14(26.92) | 0.1745 |
| Betel nut usage | 256(10.23) | 143(10.64) | 33(9.94) | 79(10.21) | 1(1.92) | 0.2423 |
| **Medication history** |  |  |  |  |  |  |
| Erythropoiesis-stimulating agents (n, %) | 550(21.28) | 280(20.5) | 98(29.43) | 156(18.73) | 16(30.77) | 0.0003 |
| Vitamin D analogue (n, %) | 164(6.35) | 85(6.22) | 28(8.41) | 48(5.76) | 3(5.77) | 0.4347 |
| Uric acid-lowering agents (n, %) | 573(22.17) | 297(21.74) | 79(23.72) | 184(22.09) | 13(25) | 0.8395 |
| Diuretics (n, %) | 1221(47.25) | 588(43.05) | 144(43.24) | 451(54.14) | 38(73.08) | <.0001 |
| pressure_diuretic_baseline | 1113(43.07) | 514(37.63) | 131(39.34) | 435(52.22) | 33(63.46) | <.0001 |
| Angiotensin converting enzyme inhibitor (n, %) | 220(8.51) | 94(6.88) | 27(8.11) | 93(11.16) | 6(11.54) | 0.0059 |
| Angiotensin II receptor blocker (n, %) | 1189(46.01) | 649(47.51) | 180(54.05) | 330(39.62) | 30(57.69) | <.0001 |
| Beta blocker (n, %) | 855(33.09) | 455(33.31) | 123(36.94) | 256(30.73) | 21(40.38) | 0.1361 |
| Calcium channel blocker (n, %) | 1339(51.82) | 703(51.46) | 185(55.56) | 413(49.58) | 38(73.08) | 0.0038 |
| Statin (n, %) | 752(29.1) | 396(28.99) | 124(37.24) | 216(25.93) | 16(30.77) | 0.0024 |
| Fibrate (n, %) | 123(4.76) | 62(4.54) | 18(5.41) | 43(5.16) | 0(0) | 0.1215 |
| Insulin: premix insulin (n, %) | 275(10.64) | 119(8.71) | 30(9.01) | 120(14.41) | 6(11.54) | 0.0004 |
| Insulin: rapid insulin (n, %) | 371(14.36) | 158(11.57) | 36(10.81) | 166(19.93) | 11(21.15) | <.0001 |
| Insulin: basal insulin (n, %) | 125(4.84) | 72(5.27) | 18(5.41) | 31(3.72) | 4(7.69) | 0.2621 |
| **Outcome** |  |  |  |  |  |  |
| Estimated glomerular filtration rate (eGFR) before 6 month | 25.9±16.3 | 27.9±17.5 | 23.8±14.6 | 23.2±14.3 | 22.4±12.2 | <.0001 |
| Estimated glomerular filtration rate (eGFR) after 6 month | 26±17.1 | 28.5±18.7 | 22.7±14.2 | 22.7±14.5 | 23.7±12.4 | <.0001 |
| Time to death (year) | 5.5±3.8 | 6.5±3.9 | 5.1±3.4 | 4.1±3.1 | 4.8±3.5 | <.0001 |
| Time to ESKD (year) | 4.4±4.2 | 4.1±4 | 3.7±3.8 | 5.2±4.6 | 4.6±3.8 | <.0001 |
| Time to death or ESKD (years) | 4.2±4.2 | 4.9±4.5 | 3.9±4.1 | 3.2±3.4 | 3.9±4 | <.0001 |
| Stage 5-CKD (2 years later) (n, %) | 712(32.26) | 348(28.62) | 118(36.2) | 231(37.44) | 15(31.25) | 0.0006 |
| Mortality (n, %) | 885(34.25) | 0(0) | 0(0) | 833(100) | 52(100) | <.0001 |
| End-stage kidney disease (ESKD) (n, %) | 1351(52.28) | 348(25.48) | 118(35.44) | 833(100) | 52(100) | <.0001 |

**2. Detailed definition of medications and mdical histories by ATC code, ICD 9 and ICD 10**

DM: ICD9=250, ICD10=E08、E09、E10、E11、E13

Hypertension:ICD9=401, ICD10= I10

Cerebrovascular disease: ICD9= 430-432、433-437, ICD10= I60、I61、I62、I63、I65、I66、I67.0- I67.7、z67.81、I67.82、I67.841、I67.848、I67.89、I68.0、I68.2、G45.0-G45.2、G45.4、G45.8、G45.9、G46.0- G46.8

Heart failure: ICD9=428.0-428.10, ICD10= I50.1- I50.4、I50.9

Hepatitis B: ICD9=070.2–070.3, ICD10=B16、B18.0、B18.1、B19.1

Hepatitis C: ICD9=070.70、070.4、070.5, ICD10=B17.0、B17.10、B17.11、B17.2、B17.8、B18.2、B18.8、B18.9、B19.20、B19.21

Acute coronary syndrome: ICD9=410-410.9、36.0-36.03、36.05-36.09、36.1-36.99、V45.81, ICD10=I21、I22、A39

Malignant dysrhythmia: ICD9=426.0、426.12-426.13、426.51-426.52、426.54、427.1、427.4、427.41-427.42、427.5, ICD10=I44.2、I44.1、I45.2、I45.3、I47.0、I47.2、I49.3、I49.01、I49.02、I46

Malginant neoplasm of liver, primary (HCC): ICD9=155.0, ICD10=C22.0、C22.2、C22.3、C22.4、C22.7、C22.8、Z51.12

Cerebral infarction: ICD9=434.01、434.11、434.91, ICD10=I63

Cerebral artery occlusion: ICD9=434.90, ICD10=I66.0、I66.1、I66.2、I66.3、I66.8、I66.9

Dementia, presenile: ICD9=290.10；ICD10=F03.90

Dementia, senile: ICD9=290.0；ICD10=F03.90

Oral Cancer : ICD9=140-146, ICD10=C00-C10、Z51.12

Nasopharyngeal cancer: ICD9=147, ICD10=C11、Z51.12

Esophageal Cancer:ICD9=150, ICD10=C15、Z51.12

Stomach Cancer: ICD9=151, ICD10= C16、C7A.092、Z51.12

Colon Cancer: ICD9=153.0-154.1, ICD10= C18.0-C18.4、C18.6- C18.7、C19-C20、C7A.020-C7A.026、Z51.12

Liver Cancer: ICD9=155, ICD10=C22.0-C22.9、Z51.12

Pancreas Cancer:ICD9=157, ICD10=C25.0-C25.9、Z51.12

Lung Cancer: ICD9=162, ICD10=C33、C34、C7A.090、Z51.12

Breast Cancer, female: ICD9=174.9, ICD10=C50.911、C50.912、C50.919、Z51.12

Endometrium Cancer: ICD9=182.0, ICD10=C54.1、C54.2、C54.3、C54.9、Z51.12

Prostate Cancer: ICD9=185, ICD10=C61、Z51.12

Bladder Cancer: ICD9=188, ICD10= C67、Z51.12

Thyroid Cancer: ICD9=193, ICD10=C73、E31.22、Z51.12

Rheumatoid arthritis: ICD9=714, ICD10=M05、M06、M12、M08

Systemic lupus erythematosus: ICD9=710.0, ICD10=M32

Hemodialysis: ICD9=585、403.01、403.11、403.91、404.02、404.03、404.12、404.13、404.92、404.93

ICD10= I12、I12.9、I13.11、I13.2、N18.4、N18.5、N18 E11.610, E11.618, E11.620, E11.621, E11.622, E11.628, E11.630, E11.638, E11.649, E11.65, E11.69.6、N18.9

Renal transpalnt: ICD9=V42.0、996.81, ICD10=D89.810-D89.813、T86.10-T86.13、T86.19、Z94.0

Medication for hypertension (including ACEI, ARB, B-blocker, Diuretics, CCB)

ACEI ATC code: 243204, ARB ATC code:243208, beta-blockerATC code: 242400, CCB ATC code:242800, and DiureticATC code: 402824, 402820, 402808

Lipid-lowering agents (includign statins, and fibrates)

Statins ATC code: 240608, and fibrates ATCcode:240606

Medication for diabetes mellitus (including Metformin, Sulfonylurea, Meglitinide, A-glucosidase inhibitor, TZD, DPP-4 inhibitor, Incretin, and SGLT-2 inhibitor)

A-glucosidase inhibitor ATC code: 682002, DPP-4 inhibitor ATC code: 682005, Incretin ATC code: 682006, Metformin ATC code: 682004, Meglitinide ATC code: 682016, Sulfonylurea ATC code: 682020, TZD ATC code: 682028, and SGLT-2 inhibitor ATC code 682018 .

Insulin: ATC code 682008

AST-120: ATC code A07BA01

Erythropoietin: ATC code BD300, AE590 and AE460

Vitamin D: ATC code DR150, ALF01 and AA200)

Uric acid-lowering agents: ATC code CC080, CA020, FEB01, AR440 and AB370

Diuretics: ATC code AD350, HYC01, AF270, AM720, AM560, AF570, CF010, CS030, AH600, SEV01, EXF01 and HYS01

Table S2. Baseline characteristics according to the usage of AST-120 or not after propensity score (1:1 matching).

|  | Overall | AST-120(-) | AST-120(+) | p-value |
| --- | --- | --- | --- | --- |
| Case number | 626 | 313 | 313 |  |
| Age (y/o) | 64.9±14.4 | 65.2±14.7 | 64.7±14.2 | 0.6576 |
| Male gender (n, %) | 381(60.86) | 188(60.06) | 193(61.66) | 0.6822 |
| Body height (cm) | 162.1±9 | 161.7±8.9 | 162.4±9 | 0.3628 |
| Body weight (kg) | 63.9±13.6 | 64.1±14.8 | 63.8±12.3 | 0.8444 |
| **Laboratory data** |  |  |  |  |
| Serum creatinine (mg/dl) | 5.2±7.9 | 5.2±7.5 | 5.2±8.3 | 0.9839 |
| Estimated glomerular filtration rate (eGFR) (ml/min/1.73m^2^) | 22.7±14.5 | 22.1±14.3 | 23.3±14.7 | 0.2956 |
| Daily proteinuria (g/day) | 3.4±8.2 | 2.8±3.3 | 4±10.6 | 0.2026 |
| Urinary albumin creatinine ratio (mg/g) | 2178.5±2542.1 | 1924.7±2297.2 | 2325.2±2673.4 | 0.337 |
| Glycated hemoglobin (HbA1c) (%) | 6.7±1.4 | 6.6±1.4 | 6.7±1.3 | 0.7491 |
| Fasting glucose (mg/dl) | 115.1±42.5 | 113.5±40.2 | 116.7±44.5 | 0.397 |
| Aspartate aminotransferase (U/L) | 25.9±21.9 | 26.4±20.6 | 25.3±23.1 | 0.6065 |
| Alanine aminotransferase (U/L) | 24.5±32.5 | 25.7±39.6 | 23.3±24.2 | 0.3873 |
| Total bilirubin (mg/dl) | 0.4±0.3 | 0.4±0.4 | 0.4±0.3 | 0.9875 |
| Total cholesterol (mg/dl) | 184.3±58.5 | 179±49.4 | 188.4±64.4 | 0.0633 |
| High-density lipoprotein (HDL) cholesterol (mg/dl) | 48.6±16.3 | 49.9±15.5 | 47.6±16.9 | 0.2994 |
| Low-density lipoprotein (LDL) cholesterol (mg/dl) | 107.8±46 | 105±37.6 | 110.1±51.8 | 0.2283 |
| Triglyceride (mg/dl) | 159.6±152.2 | 148.4±95.4 | 168.8±186.3 | 0.12 |
| Systolic blood pressure (mmHg) | 137.3±21.5 | 138.5±22.2 | 136±20.6 | 0.1913 |
| Diastolic blood pressure (mmHg) | 75.3±14.2 | 75.9±14.8 | 74.7±13.7 | 0.3377 |
| **Medical history** |  |  |  |  |
| Diabetes mellitus (n, %) | 238(38.02) | 117(37.38) | 121(38.66) | 0.7419 |
| Hypertension (n, %) | 469(74.92) | 237(75.72) | 232(74.12) | 0.6448 |
| Gout (n, %) | 167(26.68) | 90(28.75) | 77(24.6) | 0.2401 |
| Congestive heart failure (n, %) | 19(3.04) | 6(1.92) | 13(4.15) | 0.1029 |
| Ischemic heart disease (n, %) | 20(3.19) | 9(2.88) | 11(3.51) | 0.6494 |
| Cerebrovascular disease (n, %) | 28(4.47) | 17(5.43) | 11(3.51) | 0.246 |
| Liver cirrhosis (n, %) | 42(6.71) | 22(7.03) | 20(6.39) | 0.7493 |
| Malignancy (n, %) | 37(5.91) | 20(6.39) | 17(5.43) | 0.6111 |
| **Family medical history** |  |  |  |  |
| Hyperlipidemia (n, %) | 189(30.19) | 90(28.75) | 99(31.63) | 0.4333 |
| Diabetes mellitus (n, %) | 219(34.98) | 108(34.5) | 111(35.46) | 0.8015 |
| Hypertension (n, %) | 246(39.3) | 128(40.89) | 118(37.7) | 0.4132 |
| Heart disease (n, %) | 38(6.07) | 18(5.75) | 20(6.39) | 0.7378 |
| Cerebrovascular disease (n, %) | 53(8.47) | 30(9.58) | 23(7.35) | 0.3149 |
| Hyperlipidemia (n, %) | 11(1.76) | 6(1.92) | 5(1.6) | 0.761 |
| Kidney disease (n, %) | 51(8.15) | 26(8.31) | 25(7.99) | 0.8838 |
| Malignancy (n, %) | 42(6.71) | 23(7.35) | 19(6.07) | 0.5228 |
| Hereditary disease (n, %) | 3(0.48) | 2(0.64) | 1(0.32) | 0.5628 |
| Polycystic kidney disease (n, %) | 4(0.64) | 3(0.96) | 1(0.32) | 0.3158 |
| Gout (n, %) | 23(3.67) | 12(3.83) | 11(3.51) | 0.8318 |
| **Habit and physical activity** |  |  |  |  |
| Exercise: walking (n, %) | 224(35.78) | 113(36.1) | 111(35.46) | 0.8676 |
| Exercise: brisk walking (n, %) | 16(2.56) | 9(2.88) | 7(2.24) | 0.6125 |
| Exercise: running (n, %) | 12(1.92) | 7(2.24) | 5(1.6) | 0.5599 |
| Smoking (n, %) | 213(34.03) | 110(35.14) | 103(32.91) | 0.5549 |
| Alcohol drinking (n, %) | 160(25.56) | 78(24.92) | 82(26.2) | 0.714 |
| Betel nut usage | 58(9.27) | 29(9.27) | 29(9.27) | 1 |
| **Medication history** |  |  |  |  |
| Erythropoiesis-stimulating agents (n, %) | 208(33.23) | 107(34.19) | 101(32.27) | 0.6107 |
| Vitamin D analogue (n, %) | 54(8.63) | 26(8.31) | 28(8.95) | 0.7759 |
| Uric acid-lowering agents (n, %) | 162(25.88) | 84(26.84) | 78(24.92) | 0.584 |
| Diuretics (n, %) | 341(54.47) | 179(57.19) | 162(51.76) | 0.1724 |
| Angiotensin converting enzyme inhibitor (n, %) | 45(7.19) | 21(6.71) | 24(7.67) | 0.6425 |
| Angiotensin II receptor blocker (n, %) | 350(55.91) | 175(55.91) | 175(55.91) | 1 |
| Beta blocker (n, %) | 257(41.05) | 132(42.17) | 125(39.94) | 0.5695 |
| Calcium channel blocker (n, %) | 385(61.5) | 198(63.26) | 187(59.74) | 0.3662 |
| Statin (n, %) | 240(38.34) | 120(38.34) | 120(38.34) | 1 |
| Fibrate (n, %) | 28(4.47) | 14(4.47) | 14(4.47) | 1 |
| Insulin: premix insulin (n, %) | 64(10.22) | 31(9.9) | 33(10.54) | 0.7919 |
| Insulin: rapid insulin (n, %) | 86(13.74) | 41(13.1) | 45(14.38) | 0.6424 |
| Insulin: basal insulin (n, %) | 35(5.59) | 16(5.11) | 19(6.07) | 0.6017 |
| **Outcome** |  |  |  |  |
| Stage 5-CKD (2 years later) (n, %) | 244(38.98) | 127(40.58) | 117(37.38) | 0.4125 |
| Mortality (n, %) | 143(22.84) | 101(32.27) | 42(13.42) | <.0001 |
| End-stage kidney disease (ESKD) (n, %) | 333(53.19) | 186(59.42) | 147(46.96) | 0.0018 |

Table S3. Baseline characteristics according to the usage of AST-120 or not after propensity score (1:2 matching).

|  | Overall | AST-120(-) | AST-120(+) | p-value |  |
| --- | --- | --- | --- | --- | --- |
| Case number | 901 | 592 | 309 |  |  |
| Age (y/o) | 65±14.3 | 65.2±14.4 | 64.7±14.2 | 0.6514 |  |
| Male gender (n, %) | 557(61.82) | 367(61.99) | 190(61.49) | 0.8824 |  |
| Body height (cm) | 162±9.9 | 161.9±10.3 | 162.4±9.1 | 0.4497 |  |
| Body weight (kg) | 63.8±13.3 | 63.8±13.8 | 63.9±12.3 | 0.8848 |  |
| **Laboratory data** |  |  |  |  | |
| Serum creatinine (mg/dl) | 5.3±8.6 | 5.3±8.8 | 5.2±8.3 | 0.792 |  |
| Estimated glomerular filtration rate (eGFR) (ml/min/1.73m^2^) | 22.9±14.5 | 22.6±14.4 | 23.5±14.7 | 0.3631 |  |
| Daily proteinuria (g/day) | 3.3±7.1 | 2.9±3.6 | 3.9±10.6 | 0.304 |  |
| Urinary albumin creatinine ratio (mg/g) | 2055.3±2379.7 | 1803.2±2056.1 | 2350.1±2690.5 | 0.0986 |  |
| Glycated hemoglobin (HbA1c) (%) | 6.7±1.4 | 6.7±1.5 | 6.7±1.3 | 0.9052 |  |
| Fasting glucose (mg/dl) | 117.2±44.5 | 117.6±44.6 | 116.5±44.5 | 0.7423 |  |
| Aspartate aminotransferase (U/L) | 27.2±45.7 | 28.3±54.1 | 25.4±23.2 | 0.3872 |  |
| Alanine aminotransferase (U/L) | 24±28.6 | 24.3±30.8 | 23.4±24.3 | 0.67 |  |
| Total bilirubin (mg/dl) | 0.4±0.3 | 0.4±0.3 | 0.4±0.3 | 0.629 |  |
| Total cholesterol (mg/dl) | 183.1±56.9 | 179.8±51.4 | 188±64.1 | 0.0722 |  |
| High-density lipoprotein (HDL) cholesterol (mg/dl) | 49.4±16.6 | 50.4±16.3 | 47.8±17 | 0.1656 |  |
| Low-density lipoprotein (LDL) cholesterol (mg/dl) | 107.2±45.3 | 105.4±40.3 | 110.1±52 | 0.2258 |  |
| Triglyceride (mg/dl) | 155.5±141.4 | 149±112.5 | 166±178.1 | 0.1659 |  |
| Systolic blood pressure (mmHg) | 137.7±21.3 | 138.6±21.6 | 136±20.5 | 0.1166 |  |
| Diastolic blood pressure (mmHg) | 75.8±14.1 | 76.4±14.4 | 74.6±13.6 | 0.1252 |  |
| **Medical history** |  |  |  |  | |
| Diabetes mellitus (n, %) | 347(38.51) | 228(38.51) | 119(38.51) | 0.9995 |  |
| Hypertension (n, %) | 657(72.92) | 429(72.47) | 228(73.79) | 0.6721 |  |
| Gout (n, %) | 221(24.53) | 147(24.83) | 74(23.95) | 0.77 |  |
| Congestive heart failure (n, %) | 35(3.88) | 24(4.05) | 11(3.56) | 0.7155 |  |
| Ischemic heart disease (n, %) | 33(3.66) | 22(3.72) | 11(3.56) | 0.9056 |  |
| Cerebrovascular disease (n, %) | 37(4.11) | 26(4.39) | 11(3.56) | 0.5502 |  |
| Liver cirrhosis (n, %) | 52(5.77) | 33(5.57) | 19(6.15) | 0.7256 |  |
| Malignancy (n, %) | 55(6.1) | 38(6.42) | 17(5.5) | 0.5851 |  |
| **Family medical history** |  |  |  |  | |
| Hyperlipidemia (n, %) | 270(29.97) | 175(29.56) | 95(30.74) | 0.7128 |  |
| Diabetes mellitus (n, %) | 313(34.74) | 206(34.8) | 107(34.63) | 0.9596 |  |
| Hypertension (n, %) | 350(38.85) | 234(39.53) | 116(37.54) | 0.5614 |  |
| Heart disease (n, %) | 56(6.22) | 37(6.25) | 19(6.15) | 0.9524 |  |
| Cerebrovascular disease (n, %) | 74(8.21) | 51(8.61) | 23(7.44) | 0.5432 |  |
| Hyperlipidemia (n, %) | 13(1.44) | 8(1.35) | 5(1.62) | 0.7499 |  |
| Kidney disease (n, %) | 71(7.88) | 47(7.94) | 24(7.77) | 0.9274 |  |
| Malignancy (n, %) | 57(6.33) | 38(6.42) | 19(6.15) | 0.8744 |  |
| Hereditary disease (n, %) | 2(0.22) | 1(0.17) | 1(0.32) | 0.6395 |  |
| Polycystic kidney disease (n, %) | 4(0.44) | 3(0.51) | 1(0.32) | 0.6947 |  |
| Gout (n, %) | 35(3.88) | 24(4.05) | 11(3.56) | 0.7155 |  |
| **Habit and physical activity** |  |  |  |  |  |
| Exercise: walking (n, %) | 333(36.96) | 223(37.67) | 110(35.6) | 0.5411 |  |
| Exercise: brisk walking (n, %) | 19(2.11) | 12(2.03) | 7(2.27) | 0.8131 |  |
| Exercise: running (n, %) | 14(1.55) | 9(1.52) | 5(1.62) | 0.9102 |  |
| Smoking (n, %) | 317(35.18) | 214(36.15) | 103(33.33) | 0.4009 |  |
| Alcohol drinking (n, %) | 240(26.64) | 158(26.69) | 82(26.54) | 0.9609 |  |
| Betel nut usage | 89(9.88) | 60(10.14) | 29(9.39) | 0.7202 |  |
| **Medication history** |  |  |  |  | |
| Erythropoiesis-stimulating agents (n, %) | 280(31.08) | 182(30.74) | 98(31.72) | 0.7648 |  |
| Vitamin D analogue (n, %) | 78(8.66) | 52(8.78) | 26(8.41) | 0.8515 |  |
| Uric acid-lowering agents (n, %) | 226(25.08) | 148(25) | 78(25.24) | 0.9364 |  |
| Diuretics (n, %) | 484(53.72) | 324(54.73) | 160(51.78) | 0.3993 |  |
| Angiotensin converting enzyme inhibitor (n, %) | 80(8.88) | 56(9.46) | 24(7.77) | 0.3965 |  |
| Angiotensin II receptor blocker (n, %) | 497(55.16) | 324(54.73) | 173(55.99) | 0.7187 |  |
| Beta blocker (n, %) | 365(40.51) | 242(40.88) | 123(39.81) | 0.7556 |  |
| Calcium channel blocker (n, %) | 550(61.04) | 365(61.66) | 185(59.87) | 0.602 |  |
| Statin (n, %) | 346(38.4) | 227(38.34) | 119(38.51) | 0.961 |  |
| Fibrate (n, %) | 33(3.66) | 20(3.38) | 13(4.21) | 0.5296 |  |
| Insulin: premix insulin (n, %) | 90(9.99) | 58(9.8) | 32(10.36) | 0.7906 |  |
| Insulin: rapid insulin (n, %) | 135(14.98) | 90(15.2) | 45(14.56) | 0.7985 |  |
| Insulin: basal insulin (n, %) | 54(5.99) | 35(5.91) | 19(6.15) | 0.887 |  |
| **Outcome** |  |  |  |  |  |
| Stage 5-CKD (2 years later) (n, %) | 336(37.29) | 222(37.5) | 114(36.89) | 0.8581 |  |
| Mortality (n, %) | 235(26.08) | 194(32.77) | 41(13.27) | <.0001 |  |
| End-stage kidney disease (ESKD) (n, %) | 478(53.05) | 334(56.42) | 144(46.6) | 0.0051 |  |

Table S4. The optimized parameters in the XGboost and DNN.

**-----------------------------------------------------------------------------------------------------------------------**

**XGBoost**

base_score=0.5, booster='gbtree', colsample_bylevel=1, colsample_bytree=1,

gamma=0, learning_rate= 0.1, max_depth=6, min_child_weight=1,

n_estimators= 100, subsample=1,max_delta_step=0.0, n_jobs=1,

objective='binary:logistic',random_state=0, reg_alpha=0,

reg_lambda=1.0, scale_pos_weight=1.0, tree_method='exact'

**DNN**

In DNN, the deep neural network's structure was designed as follows: 46 input layers → 46 middle hidden layers → 46 middle hidden layers → 46 middle hidden layers → one-dimensional output layer, with the binary outcome of death set as the output layer. To prevent overfitting during model training in deep learning, we incorporated a dropout layer between the hidden layers with a dropout rate of 0.3. We used scaled exponential linear units as activation functions in the middle layers and hard sigmoid units in the output layer.

------------------------------------------------------------------------------------------------------------------------

Figure S1. Schoenfeld residuals versus time for HospiceReferral to validate the assumption.


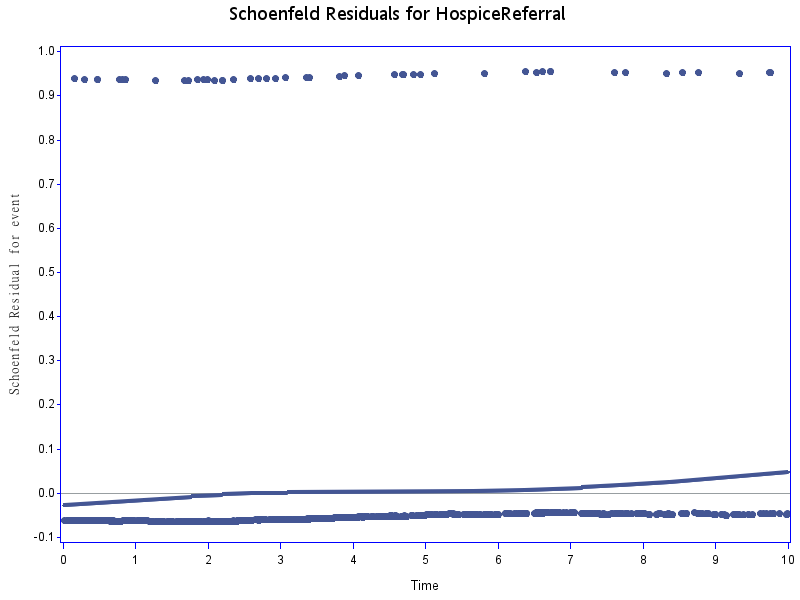


Figure S2. Algorithm for patients’ selection for analysis.


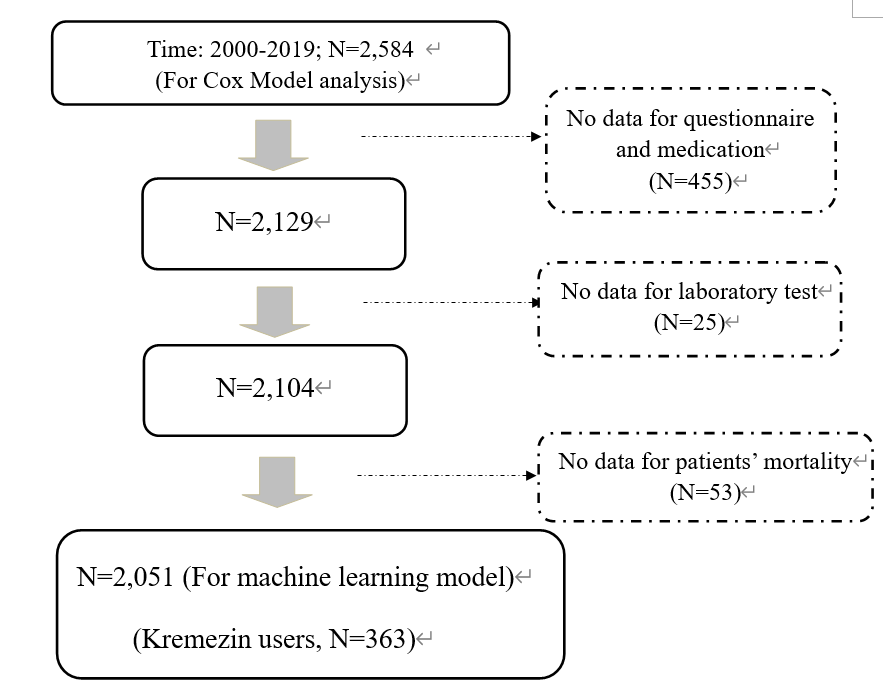

Supplement: Supplementary file 1 — Supplementary Information. [file 41598_2024_51498_MOESM1_ESM.docx]
